# Local and Global Feature-Aware Dual-Branch Networks for Plant Disease Recognition

**DOI:** 10.34133/plantphenomics.0208

**Published:** 2024-07-31

**Authors:** Jianwu Lin, Xin Zhang, Yongbin Qin, Shengxian Yang, Xingtian Wen, Tomislav Cernava, Quirico Migheli, Xiaoyulong Chen

**Affiliations:** ^1^ Text Computing & Cognitive Intelligence Engineering Research Center of National Education Ministry, College of Computer Science and Technology, Guizhou University, Guiyang 550025, China.; ^2^ State Key Laboratory of Public Big Data, College of Computer Science and Technology, Guizhou University, Guiyang 550025, China.; ^3^ College of Big Data and Information Engineering, Guizhou University, Guiyang 550025, China.; ^4^ Guizhou-Europe Environmental Biotechnology and Agricultural Informatics Oversea Innovation Center in Guizhou University, Guizhou Provincial Science and Technology Department, Guiyang 550025, China.; ^5^ School of Biological Sciences, Faculty of Environmental and Life Sciences, University of Southampton, Southampton S017 1BJ, UK.; ^6^ Dipartimento di Agraria and NRD—Nucleo di Ricerca sulla Desertificazione, Università degli Studi di Sassari, Sassari, Italy.; ^7^ College of Life Sciences, Guizhou University, Guiyang 550025, China.

## Abstract

Accurate identification of plant diseases is important for ensuring the safety of agricultural production. Convolutional neural networks (CNNs) and visual transformers (VTs) can extract effective representations of images and have been widely used for the intelligent recognition of plant disease images. However, CNNs have excellent local perception with poor global perception, and VTs have excellent global perception with poor local perception. This makes it difficult to further improve the performance of both CNNs and VTs on plant disease recognition tasks. In this paper, we propose a local and global feature-aware dual-branch network, named LGNet, for the identification of plant diseases. More specifically, we first design a dual-branch structure based on CNNs and VTs to extract the local and global features. Then, an adaptive feature fusion (AFF) module is designed to fuse the local and global features, thus driving the model to dynamically perceive the weights of different features. Finally, we design a hierarchical mixed-scale unit-guided feature fusion (HMUFF) module to mine the key information in the features at different levels and fuse the differentiated information among them, thereby enhancing the model's multiscale perception capability. Subsequently, extensive experiments were conducted on the AI Challenger 2018 dataset and the self-collected corn disease (SCD) dataset. The experimental results demonstrate that our proposed LGNet achieves state-of-the-art recognition performance on both the AI Challenger 2018 dataset and the SCD dataset, with accuracies of 88.74% and 99.08%, respectively.

## Introduction

Safeguarding the agricultural production process has become an important economic imperative and is aligned with the growing demand for improved quality and improved yields of agricultural products. Plant diseases are among the most important factors threatening the security of agricultural production [1]. At present, pests and diseases have led to a decline in the yields of a large number of crops [2]. Precise management of plant diseases could substantially reduce crop losses in the future. Accurate identification of plant diseases is a prerequisite for precision prevention and control [3–5]. However, traditional methods of plant disease identification, which are usually based on farmers’ long-standing experience, are time consuming and have difficulty meeting the demands of large-scale cultivation [6–9].

In recent years, image processing technology has developed rapidly, and image-based plant disease recognition tasks have become popular [4,10–15]. For example, identifying plant diseases using traditional machine learning methods achieved good performance in the early days [16–19]. However, this method usually requires manual feature extractions, making the recognition accuracy low when coping with large-scale plant diseases or plant diseases in the field environment. With the rapid development of artificial intelligence, deep learning, with its strong feature representation capabilities, has been increasingly applied for image recognition tasks, such as image classification [20–22], object detection [23–25], and image segmentation [26–28]. Currently, deep learning-based methods, which can be categorized into convolutional neural network (CNN)-based methods and visual transformer (VT)-based methods, have become the mainstream methods for identifying plant diseases [29–31]. The CNN-based method uses a sliding window to extract features from plant disease images and therefore has excellent local feature perception with poor global feature perception [32–34]. The VT-based method transforms the image into multiple patches and then uses multi-head self-attention to extract the features of the plant disease image and therefore has excellent global feature perception with poor local feature perception [11,35,36]. However, some of the existing state-of-the-art (SOTA) methods for plant disease identification are based on a single CNN or VT, which severely limits their performance [10,35,37–42]. This is because the symptoms exhibited by plant diseases are usually diverse, i.e., some plants are less infested with the pathogen, so the spots are localized, while some plants are more infested with the pathogen, so the spots are global, thus making it impossible for a single CNN or VT to strike a balance between local and global feature extraction. Although fusion architectures based on CNNs and VTs have been proposed, they still ignore the weights between features from different architectures [43].

To address the above challenge, we propose a local and global feature-aware dual-branch network, named LGNet, for plant disease recognition. Specifically, to extract both local and global disease features, we develop a hybrid 2-branch network based on a CNN and VT. In addition, we design an adaptive feature fusion (AFF) module and a multilevel feature fusion module for local and global disease feature perception and multiscale feature fusion, respectively. LGNet achieves SOTA performance on 2 plant disease datasets. Our contributions are as follows:

1. We propose a hybrid dual-branch network based on a CNN and VT for plant disease recognition.

2. An AFF module is designed for the adaptive perception of local and global features.

3. We design a hierarchical mixed-scale unit-guided feature fusion (HMUFF) module to mine effective information in features and fuse it at multiple scales.

4. Our proposed LGNet achieves SOTA performance on the AI Challenger 2018 dataset and the self-collected corn disease (SCD) dataset.

### Related work

#### Traditional machine learning methods

Recognizing plant diseases using traditional machine learning methods can be classified in 3 steps: image preprocessing, feature design, and classification using the obtained features [44]. Omrani et al. [45] used k-means to detect diseased areas in apple images and utilized a support vector machine (SVM) for classification. Rumpf et al. [17] used SVM and hyperspectral techniques to achieve early disease identification in sugar beets. Experiments showed that their method reached an accuracy of 97%. Phadikar et al. [46] selected rice disease features using rough set theory (RST). The experimental results showed that the RST method outperformed traditional methods in terms of feature selection. Plant disease identification using traditional machine learning methods usually has strong subjectivities and poor generalization abilities and has been gradually replaced by deep learning-based methods.

#### Deep learning-based methods

Unlike traditional methods, deep learning-based methods can automatically extract effective features of plant diseases and therefore have stronger generalizability. Nawaz et al. [47] proposed an improved CenterNet for coffee plant leaf disease recognition by introducing an improved ResNet-50. The accuracy and mean average precision (map) of their proposed model were 98.54% and 97%, respectively. Salamai et al. [11] developed a lesion-aware VT for paddy leaf disease detection. Their model achieved an average accuracy of 98.74% and an average f1-score of 98.18% on public paddy disease datasets. Thai et al. [48] proposed the least important attention pruning (LeIAP) algorithm to improve the transformer model. Furthermore, they also used sparse matrix–matrix multiplication (SPMM) to calculate matrix correlations. Their developed model was more accurate and had a smaller number of parameters than the other models. Moreover, Faisal et al. [49] developed DFNet for plant disease classification by using a double-pretrained CNN model. The DFNet model achieved 97.53% and 94.65% accuracy on the 2 datasets, respectively. The abovementioned studies verified the feasibility of the deep learning-based methods. However, almost all these methods are based on a single CNN or VT. These methods are limited in their performance when applied to diverse plant disease datasets. For this reason, we develop a hybrid architecture based on a CNN and VT to address this challenge.

## Materials and Methods

### Dataset

#### AI Challenger 2018 dataset

**Fig. 1.**
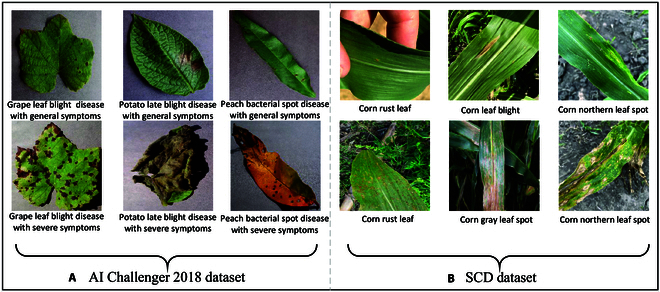
(A and B) Representative examples from the AI Challenger 2018 and SCD datasets.

The AI Challenger 2018 dataset is an open-source large-scale plant disease dataset containing a total of 36,258 plant disease images, of which 32,660 are in the training set and 3,598 are in the test set. The dataset can be categorized into 10 categories by disease type and 61 categories by disease severity. Representative samples are shown in Fig. 1A. Plant diseases with severe symptoms exhibit global symptoms, and plant diseases with general symptoms exhibit local symptoms. In addition, we further divided the training set into a new training set and a validation set at a ratio of 9:1. Thus, 29,394 images are used for model training, 3,266 images are used for model validation, and 3,598 images are used for testing the final performance of the model.

#### SCD dataset

The SCD dataset is available from 4 sources, namely, CD&S [50], PlantDoc [51], and websites. More specifically, we collected images of northern leaf blight, gray leaf spot, and northern leaf spot from the CD&S dataset, and corn embroidery disease images from PlantDoc. In addition, we obtained images of healthy corn and some diseased plants from the website. The SCD dataset contained a total of 3,258 images of 6 corn leaf diseases. An 8:1:1 ratio is randomly used for division to obtain 2,609 images in the training set, 324 images in the validation set, and 325 images in the test set. Representative samples of the SCD dataset are shown in Fig. 1B. Corn leaf diseases are characterized by both local and global symptoms and are affected by complex conditions.

### Proposed LGNet

**Fig. 2.**
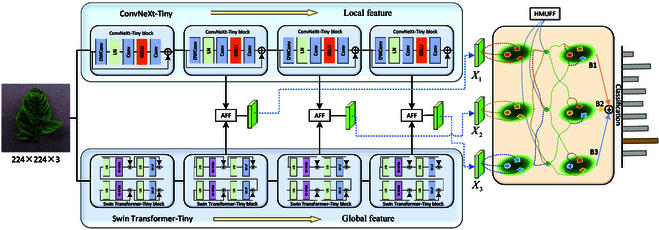
The overall architecture of LGNet consists of a dual-branch backbone network, AFF modules, and HMUFF modules.

Figure 2 shows the overall structure of LGNet, which consists of a dual-branch backbone network, AFF modules, and HMUFF modules. Specifically, we first crop the size of the input image to 224 × 224. Then, a dual-branch backbone network is used for multiscale feature extraction. In the dual-branch backbone network, we use ConvNext-Tiny [52] to extract the multiscale local features and Swin Transformer-Tiny [53] to extract the multiscale global features. Subsequently, the AFF modules are designed to achieve adaptive perceptual fusion of the local and global features, driving the model to dynamically perceive the local and global features. In addition, the HMUFF modules are designed to extract vital information from the features to guide efficient fusions between the multiscale features. More specifically, the HMUFF module mines differential features between different layers and then fuses them, thus providing each layer with a strong representation. Finally, the 3 scales of feature representations are fed into the pooling layers and classifiers to complete the identification of the plant disease.

#### AFF module

**Fig. 3.**
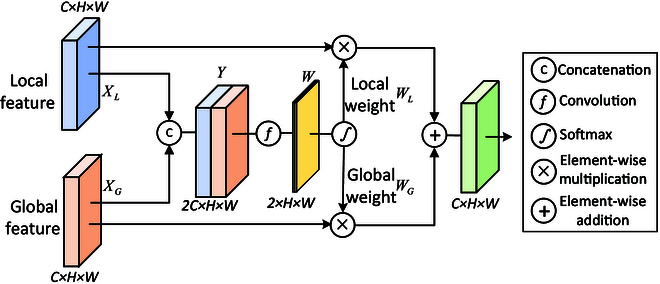
Structure of the AFF module.

Usually, CNNs can capture local disease information, and VTs can capture global disease information. However, the diverse symptom representations of plant diseases make it necessary for the model to learn more appropriate feature representations for different plant diseases. To address this challenge, as described in the previous section, we designed a hybrid 2-branch backbone network to extract the local and global features of plant diseases. Therefore, an efficient fusion module for fusing local and global features is needed. Here, we design the AFF module to implement the adaptive perceptual fusion of the local and global features, allowing the model to adaptively adjust the weights of the different features. Figure 3 illustrates the structure of the AFF module, which learns the adaptive weights of the 2 features and then weights the obtained weights on the original feature maps, enabling the model to adaptively perceive the local and global disease features, thus enhancing the model’s feature representation capability.

Given a local feature input *X_L_* ∈ ℝ^*C* × *W* × *H*^ and a global feature input *X_G_* ∈ ℝ^*C* × *W* × *H*^, *C*, *W*, and *H* denote the channel number, width, and height of the feature map, respectively. First, we cascade the 2 feature maps (*X_L_* and *X_G_*) along the channel direction to obtain the input feature *Y*. It can be written as:

$$Y=\text{Concat}\left\{X_{L};X_{G}\right\}$$ (1)

where *Y* ∈ ℝ^2*C* × *W* × *H*^. Then, we map the number of channels of the output to 2 via a convolution operation and use softmax to obtain the weighted feature map *W*. It can be written as:

$$W=\text{Softmax}\left(f_{3\times 3}\left(Y\right)\right)$$ (2)

where *f*_3 × 3_ denotes a 3 × 3 convolution. Subsequently, we separate the weight feature map *W* along the channel directions to obtain the local weights *W_L_* and global weights *W_G_*. Finally, we apply the obtained weights to the original feature map and perform elementwise addition to obtain the adaptive output *A*:

$$A={X_{L}}^{*}W_{L}+{X_{G}}^{*}W_{G}$$ (3)

#### HMUFF module

**Fig. 4.**
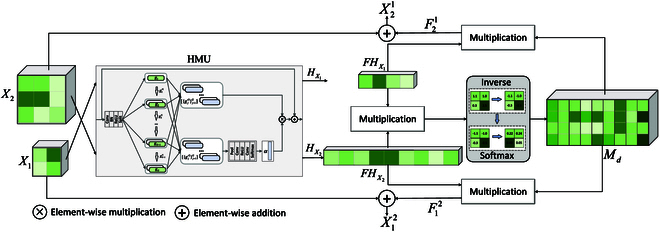
Structure of the HMUFF module.

In general, different scales of feature maps contain different key information, so fusing the multiscale features facilitates the performance of the model. In LGNet, we design 3 branches in the backbone network to extract 3 scales of feature maps. Therefore, an efficient feature fusion method is needed for fusing feature information at these 3 scales. Here, we design an HMUFF module for the fusion of different scale features. Specifically, the key ground information in the different scale features is first extracted by the hierarchical mixed-scale unit (HMU) module, thus guiding the well-designed module to realize the fusion of the multiscale features. As shown in Fig. 4, an HMU module is first used to mine key information in the feature map, and then a feature fusion module is employed to fuse the differential features between the different scales.

Given 2 input feature maps *X*_1_ ∈ ℝ^*C* × *W*_1_ × *H*_1_^ and *X*_2_ ∈ ℝ^*C* × *W*_2_ × *H*_2_^, where *C*, *W*, and *H* denote the channel number, width, and height of the input feature map, respectively. First, we use an HMU to identify and highlight key disease features:

$$H_{X_{1}}=\mathrm{HMU}\left(X_{1}\right)$$ (4)

$$H_{X_{2}}=\mathrm{HMU}\left(X_{2}\right)$$ (5)

Then, *H*_*X*_1__ and *H*_*X*_2__ are flattened through the spatial direction to obtain *FH*_*X*_1__ ∈ ℝ^*C* × *S*_1_^ and *FH*_*X*_2__ ∈ ℝ^*C* × *S*_2_^. Here, *S*_1_ = *W*_1_ ∗ *H*_1_ and *S*_2_ = *W*_2_ ∗ *H*_2_. Subsequently, we obtain the differentiated feature matrix of the 2 feature maps via matrix multiplication, inverse, and softmax operations:

$$\begin{array}{c} M_{d}=\text{Softmax}\left(-Z\left(\text{Tanspose}\left({\mathrm{FH}}_{X_{1}}\right),{\mathrm{FH}}_{X_{2}}\right)\right) \end{array}$$ (6)

where *Z* denotes matrix multiplication. Next, we perform matrix multiplication to obtain the fusion information corresponding to the feature map:

$$\begin{array}{c} F_{1}^{2}=\text{Reshape}\left(Z\left(M_{d},\text{Tanspose}\left({\mathrm{FH}}_{X_{2}}\right)\right)\right) \end{array}$$ (7)

$$F_{2}^{1}=\text{Reshape}\left(Z\left(M_{d},{\mathrm{FH}}_{X_{1}}\right)\right)$$ (8)

where $F_{1}^{2}$ denotes the fusion information corresponding to *X*_1_, and $F_{2}^{1}$ denotes the fusion information corresponding to *X*_2_. Therefore, the output of the HMUFF module is as follows:

$$X_{1}^{2}=X_{1}+F_{1}^{2}$$ (9)

$$X_{2}^{1}=X_{2}+F_{2}^{1}$$ (10)

The HMU module was originally proposed in the field of camouflaged object detection to mine the discriminative semantic features of camouflaged objects [32]. In this study, we design 3 multiscale branches based on a hybrid backbone network, as shown in Fig. 2. Therefore, we first use the HMU module to refine the multiscale features and mine the discriminative feature information in different branches. For example, mining detailed information such as textures and edges in shallow features, and semantic information in deep features, can guide the feature fusion module for efficient multiscale feature fusion. As shown in Fig. 4, the structure of the HMU module can be divided into 2 stages, namely, groupwise iteration and channelwise modulation. For the groupwise iteration stage, a convolution operation is first used to expand the number of channels of the input feature map *X*. Then, we divide the feature maps into *G* groups ${\left\{g_{j}\right\}}_{j=1}^{G}$ along the channel direction. Next, we divide the first group {*g_j_*} into 3 parts ${\left\{{g^{\prime }}_{1}^{k}\right\}}_{k=1}^{3}$ using the convolution operation. The first part, ${g^{\prime }}_{1}^{1}$, is used for feature interaction with the next group, and the other 2 parts are used for channel modulation. With this continuous iterative approach, the key feature information in the channel is mined, enabling the module to be more discriminative with the semantic information. For the channelwise modulation stage, the features $\left[{\left\{{g^{\prime }}_{j}^{2}\right\}}_{j=1}^{G}\right]$ are nonlinearly transformed to obtain the weights *α*, which are then weighted on the features $\left[{\left\{{g^{\prime }}_{j}^{3}\right\}}_{j=1}^{G}\right]$ to further highlight the key information in the features. Finally, the output *H* of the HMU module can be written as:

$$H=A\left(X+N\left(T\left(\alpha \cdot \left[{\left\{{g^{\prime }}_{j}^{3}\right\}}_{j=1}^{G}\right]\right)\right)\right)$$ (11)

**Fig. 5.**
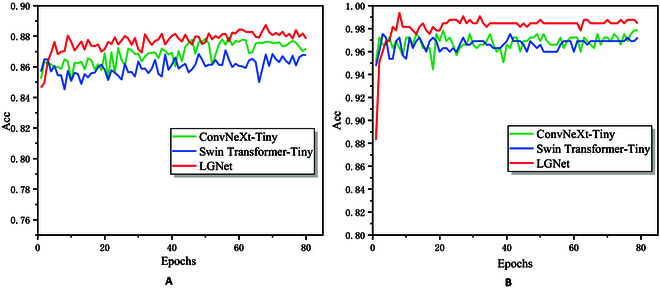
The accuracy of each epoch. (A) Results on the AI Challenger 2018 dataset. (B) Results on the SCD dataset.

where A represents the activation function, N represents the normalization layer, and T represents the convolution operation.

## Results

### Experimental environment and evaluation indices

We divided the parameters of LGNet into 2 parts for training. First, we initialized the weights of the dual-branch backbone network using the official model weights on ImageNet 1k. Therefore, for the parameters of the backbone network, we simply needed to fine-tune them using an initial learning rate of 2e^−5^. On the other hand, for parameters that were not loaded with the corresponding pretrained weights, we used an initial learning rate of 2e^−4^. SGD was adopted as the optimizer with momentum = 0.9 and weight decay = 5e^−6^. The batch size and number of iterations were set to 32 and 80, respectively. During the training process, we used online data augmentation with random rotation angles and random horizontal flips to enhance the generalization ability of the model. The cross-entropy function was used as the loss function for the 3 branches in the LGNet model. For all the experiments, we carried out training on a Windows 11 system with an NVIDIA GeForce RTX 3090 GPU and PyTorch.

For the quantitative evaluation of models, the evaluation indices used in this study are as follows:

$$\mathrm{Acc}=\frac{\mathrm{TP}+\mathrm{TN}}{\mathrm{TP}+\mathrm{FP}+\mathrm{TN}+\mathrm{FN}}$$ (12)

$$\mathrm{Pre}=\frac{\mathrm{TP}}{\mathrm{TP}+\mathrm{FP}}$$ (13)

$$\mathrm{Rec}=\frac{\mathrm{TP}}{\mathrm{TP}+\mathrm{FN}}$$ (14)

$$F1=\frac{2\mathrm{TP}}{2\mathrm{TP}+\mathrm{FP}+\mathrm{FN}}$$ (15)

where *TP* denotes the number of true-positive samples, *FP* denotes the number of false-positive samples, *FN* denotes the number of false-negative samples, and *TN* denotes the number of true-negative samples.

### Comparisons with single models

To verify the better performance of LGNet compared to a single deep learning model, we conduct comparison experiments using ConvNeXt-Tiny and Swin Transformer-Tiny (the backbone networks in LGNet). Figure 5 illustrates the accuracy of each epoch on the validation set. All 3 models are initialized with pretraining weights during the training process, so the accuracy at the beginning of training can also be maintained at a high level. For example, the accuracy of the first epoch of all 3 models on the AI Challenger 2018 dataset is greater than 84%, and the accuracy of the first epoch of all 3 models on the SCD dataset is greater than 88%. However, more parameters need to be fine-tuned during the training of the LGNet model; thus, the initial accuracy of LGNet is slightly lower than that of the other 2 single models. More importantly, when the model converges to fit, LGNet’s accuracy substantially improves, with its accuracy being approximately 1 to 2% higher than that of the single models on both datasets. These findings validate that LGNet has a more powerful feature extraction capability compared to a single model.

Table 1 illustrates the comparison results with single deep learning models on the test set. For the AI Challenger 2018 dataset, the recognition accuracies of LGNet are 1.34% and 1.65% higher than those of ConvNeXt-Tiny and Swin Transformer-Tiny, respectively. For the SCD dataset, the recognition accuracy of LGNet is 1.55% and 1.88% higher than that of ConvNeXt-Tiny and Swin Transformer-Tiny, respectively. These results are almost the same as those analyzed on the validation set, further validating that our proposed LGNet has better performance advantages for plant disease identification tasks.

**Table 1.** Comparison results with single deep learning models on the test set

Model	Acc (%)	
	AI Challenger 2018 dataset	SCD dataset
ConvNeXt-Tiny	87.4	97.53
Swin Transformer-Tiny	87.09	97.2
LGNet	88.74	99.08

### Ablation analysis

To validate the impact of the AFF modules and the HMUFF modules on the performance of the model, we perform ablation experiments on the AI Challenger 2018 and SCD datasets. Table 2 exhibits the comparison results of the ablation experiments. Both the use of the AFF modules and the use of the HMUFF modules improved the performance. In contrast, using the AFF modules on the benchmark model provides a better performance improvement than using the HMUFF modules on the benchmark model, with accuracies of 88.33% and 98.46% on the AI Challenger 2018 and SCD datasets, respectively. This may be because the dual-branch backbone network extracts rich disease features, and the AFF module allows LGNet to adaptively extract key global and local features, whereas the model using only the HMUFF module extracts a large amount of redundant information, which limits the performance of the model. Overall, the model that includes both the AFF modules and the HMUFF modules achieves a SOTA performance in most of the metrics, with accuracies of 88.74% and 99.08% on the AI Challenger 2018 and SCD datasets, respectively, which are 0.77% and 1.23% better than those of the benchmark model.

**Table 2.** Comparison of the results of ablation experiments

AFF	HMUFF	AI Challenger 2018 dataset				SCD dataset			
		Acc (%)	Pre (%)	Rec (%)	F1 (%)	Acc (%)	Pre (%)	Rec (%)	F1 (%)
		87.97	84.03	82.08	82.68	97.85	97.85	97.76	97.79
✓		88.33	85.71	84.03	84.55	98.46	98.6	98.48	98.53
	✓	88.07	82.59	84.38	82.97	98.15	98.23	98.18	98.18
✓	✓	88.74	85.58	84.68	84.94	99.08	99.31	99.18	99.23

**Fig. 6.**
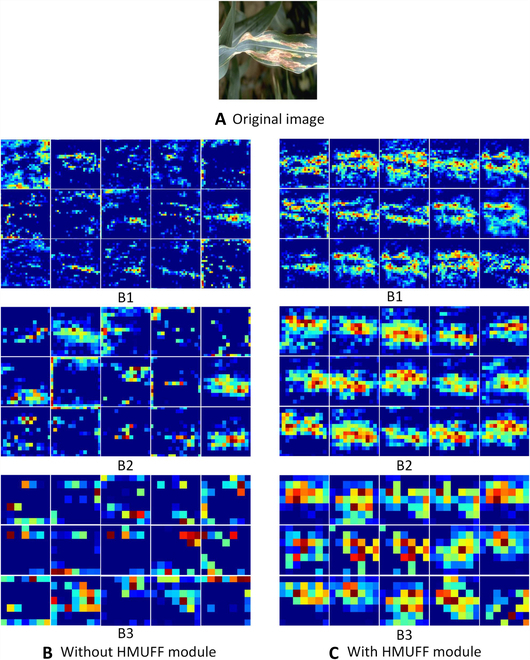
Visualization of feature maps for different branches. (A) Original image. (B) Model without the HMUFF module. (C) Model with HMUFF modules.

To show the effectiveness of the HMUFF modules, we visualize the feature maps of the 3 branches in LGNet. The visualization results are shown in Fig. 6. B1, B2, and B3 represent the 3 scales of the branches. The model with the HMUFF module not only captures more detailed texture information but also retains richer disease information than the model without the HMUFF module. On the one hand, the HMU module in the HMUFF module is able to mine more detailed texture information; on the other hand, we utilize the differentiated matrix to fuse features at different scales, which enables different branches to fuse key information they do not have; therefore, the obtained feature maps are richer in disease information. These findings demonstrate the effectiveness of our designed HMUFF module.

**Fig. 7.**
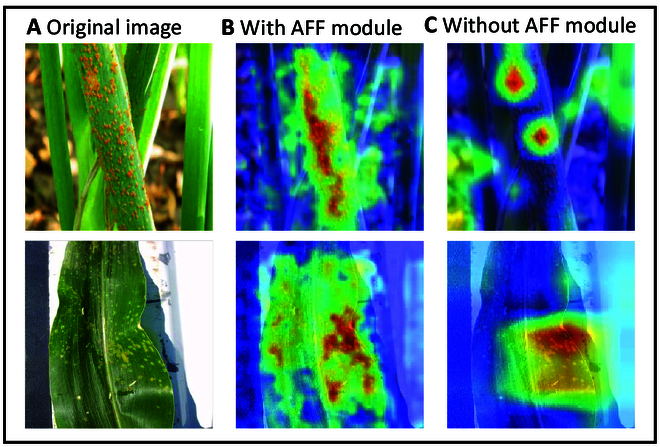
Class-activation mapping of the 2 models. (A) Original image. (B) Model without AFF modules. (C) Model with AFF modules.

The AFF module is designed to adaptively fuse the weights of the local and global features. Therefore, we validate the effectiveness of the AFF module using Grad-cam [54]. Figure 7 illustrates the obtained results. The model with the AFF module is able to localize the lesion area better and has a strong lesion perception ability. The model without the AFF module can only focus on part of the lesion area and has a poor lesion perception ability. These findings demonstrate the effectiveness of our designed AFF module.

### Impact of multiscale branches on LGNet

As shown in Fig. 2, we use the multiscale features in LGNet to enable the model to learn features of different granularities. Specifically, there are 3 scales of branches. Therefore, to verify the impact of the multiscale branches on LGNet, we conduct comparative experiments using different combinations of the 3 branches, and the results on the test set are shown in Table 3. The performance of the combination “B1 + B3” is slightly better than that of the combination “B2 + B3.” This may be because the B1 branch represents shallow features, which can provide a more subtle feature representation. In addition, both the “B1 + B3” and “B2 + B3” combinations had greater recognition performance than did the “B1 + B2” combination, which indicates the greater importance of the F3 branch in the disease recognition task. Finally, the combination of B1 + B2 + B3 achieves the highest performance for most of the metrics, with accuracies of 88.74% and 99.08% on the AI Challenger 2018 and SCD datasets, respectively. These findings demonstrate the effectiveness of using the multiscale branches on LGNet.

**Table 3.** Experimental results using the multiscale branches on the test set

	AI Challenger 2018 dataset				SCD dataset			
	Acc (%)	Pre (%)	Rec (%)	F1 (%)	Acc (%)	Pre (%)	Rec (%)	F1 (%)
B1 + B2	87.99	84.76	83.26	83.72	98.15	98.23	98.18	98.18
B2 + B3	88.52	84.92	83.82	84.1	98.46	98.46	98.45	98.44
B1 + B3	88.55	86.08	84.38	84.91	98.77	98.93	98.87	98.88
B1 + B2 + B3	88.74	85.58	84.68	84.94	99.08	99.31	99.18	99.23

### Visualization analysis

**Fig. 8.**
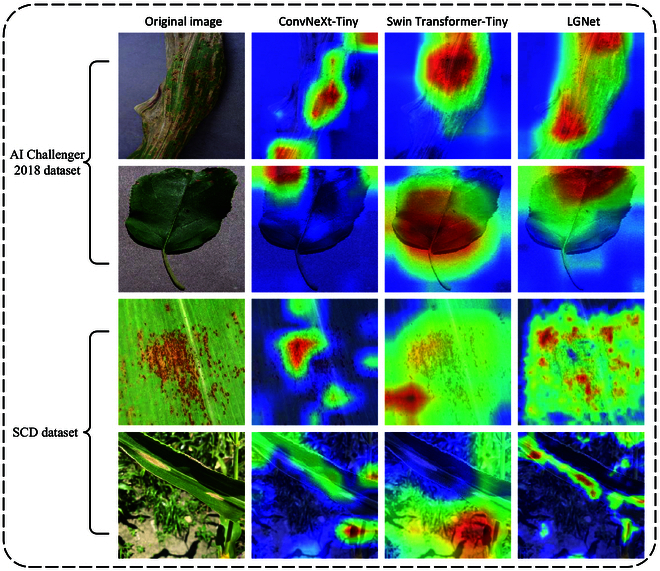
Class activation visualization results on the AI Challenger 2018 dataset and SCD dataset.

In this section, we visualize the class activation mapping for some of the samples using Grad-CAM to demonstrate the regions of interest for different models. As shown in Fig. 8, we chose the 2 samples with local disease features and global disease features from the AI Challenger 2018 dataset and SCD dataset, respectively. It can be seen that ConvNeXt-Tiny focuses on inaccurate or incomplete lesion areas and has weak lesion feature perception in complex scenes. Although Swin Transformer-Tiny can localize the lesion area, it also focuses on a large amount of redundant information. In contrast, our proposed LGNet not only focuses on global disease features but also accurately captures local features while suppressing complex background information.

**Fig. 9.**
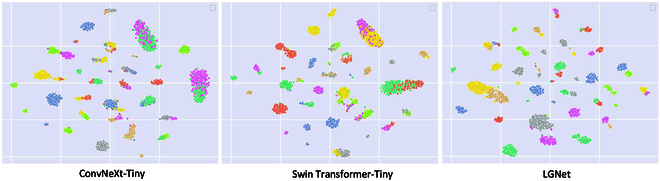
The t-SNE visualization results for the 3 models on the AI Challenger 2018 dataset.

To demonstrate the intra- and interclass distances in the test samples, we extract the last layer of the model and use t-distributed stochastic neighbor embedding (t-SNE) [55] to reduce the test set on the AI Challenger 2018 dataset to 2 dimensions. The t-SNE visualization results for the 3 models are shown in Fig. 9. All 3 models can effectively distinguish between different disease types. However, for subtypes of the same disease, it is usually difficult for ConvNext-Tiny and Swin Transformer-Tiny to categorize them accurately. In contrast, LGNet not only implements precise classifications between disease categories but also improves the metric distance between different subcategories. This is because LGNet can adaptively perceive different symptomatic disease areas and introduces well-designed modules to extract key features, thus resulting in stronger feature representations.

### Comparisons with SOTAs on the AI Challenger 2018 dataset

The comparison results between our proposed LGNet and the SOTA models on the AI Challenger 2018 dataset are shown in Table 4. LGNet achieves a SOTA performance with an accuracy of 88.74%. In contrast, previous studies have almost always been designed based on a single CNN or VT model, making their performances limited to diverse plant disease datasets. Although the hybrid structure of CNN and VT is also used in the ConvVIT-Ti+ model, they simply fused the structure of the convolutional structure and the structure of the self-attention module and did not consider the trade-off between the global disease features and the local disease features. This makes their accuracy only 86.17%, which is 2.57% lower than that of LGNet, verifying that our proposed hybrid architecture based on a CNN and VT is effective.

**Table 4.** Comparison results on the AI Challenger 2018 dataset

Model	Acc (%)
Inception-ResNet-v2 [58]	86.1
DECA_ResNet [30]	86.35
Swin Transformer-Tiny [53]	87.09
ConvViT-Ti+ [43]	86.17
ConvNeXt-Tiny [52]	87.4
SMLP_ResNet [59]	86.93
Improved attention model [60]	87.11
HCNet [61]	88.42
LGNet	88.74

### Comparisons with SOTAs on the SCD dataset

We verify that LGNet also has a SOTA performance on the plant disease recognition task in a field environment. We conduct comparative experiments on the SCD dataset using several classical models and several previous corn disease recognition models. Table 5 exhibits the comparison results on the SCD dataset. LGNet achieves the highest recognition accuracy of 99.08%, which is 0.61% and 0.31% higher than that of DFCANet and FCA-EfficientNet, respectively, further demonstrating the effectiveness of our proposed method.

**Table 5.** Comparison results on the SCD dataset

Model	Acc (%)
ResNet50 [62]	96.92
MobileNetV2 [63]	96.3
Swin Transformer-Tiny [53]	97.2
ConvNeXt-Tiny [52]	97.53
DFCANet [64]	98.47
FCA-EfficientNet [65]	98.77
LGNet	99.08

## Discussion

We propose a dual-branch network, LGNet, based on a CNN and VT for plant disease recognition. In LGNet, the AFF module is designed to efficiently fuse CNNs and VTs for local and global feature extraction of plant diseases. In addition, we design the HMUFF module to fuse multiscale disease features to further enhance the disease sensing capabilities. Subsequently, extensive experimental results validate that our method is more effective than a single deep network, and it outperforms the existing SOTA plant disease recognition models on both the AI Challenger 2018 dataset and the SCD dataset.

Moreover, through our study, we found that there are still some key issues that need to be addressed, such as efficient plant disease recognition models and robust plant disease recognition models. We further discuss them in the following subsection.

### Efficient plant disease recognition models

Efficient plant disease recognition models are those that can accurately identify various plant diseases while maintaining low computational complexity, fast inference speeds, and minimal resource utilizations. In agricultural production environments, the development of efficient plant disease recognition models is particularly important due to the scarcity of computing power and poor equipment. Given that LGNet is designed based on the parallel structure of CNN and VT, the number of parameters and computations of LGNet will also increase substantially. Therefore, the development of efficient models using machine learning methods is an important direction for our future studies [56]. Knowledge distillation could be an effective means to solve this problem. Knowledge distillation aims to transfer knowledge from a larger, high-performing teacher model to a smaller, more efficient student model that can help retain the recognition capabilities of the former while inheriting the compactness of the latter [57]. In the future, we will develop LGNet with a larger backbone network (ConvNeXt-Base and Swin Transformer-Base) to gain more in-depth knowledge and then use it as a teacher model to guide lightweight student models to learn more effective representations, thus designing highly efficient disease recognition models that can be deployed on mobile devices to help users identify plant diseases.

### Robust plant disease recognition models

In real agricultural environments, plant diseases not only have complex backgrounds but also suffer from other environmental factors, such as light, rain, and fog. This is a challenge for existing deep learning models. From the dataset perspective, obtaining more samples from real environments or mimicking real environments for data augmentation is extremely effective. From a model structure perspective, in general, large-size models have more complex network results and therefore greater robustness. In addition, model training using the current SOTA training methods, such as the meta-learning-based methods and self-supervised learning-based methods, can improve the robustness of the model. Overall, the development of robust plant disease recognition models, and improving the generalization ability of these models in real-world environments, is highly important for agricultural production.

## Data Availability

The experimental results obtained in this study are available from the corresponding author.
